# A Commemorative Special Issue in Honor of Professor Victor Nikonenko

**DOI:** 10.3390/membranes13090774

**Published:** 2023-09-01

**Authors:** Lasâad Dammak, Natalia Pismenskaya

**Affiliations:** 1 Institut de Chimie et des Matériaux Paris-Est (ICMPE), Université Paris-Est (UPEC), UMR 7182, CNRS, 2–8 rue Henri Dunant, 94320 Thiais, France; dammak@u-pec.fr; 2 Membrane Institute, Kuban State University, Krasnodar 350040, Russia

Victor Nikonenko is celebrating his 70th birthday this year. He graduated from the Faculty of Mathematics at Kuban State University, Russia in 1975 and then received his PhD (Kuban State University, Krasnodar, Russia); DSc (Institute of Electrochemistry, RAS, Moscow, Russia); as well as his Diplôme d’Habilitation à Diriger des Recherches (University Paris-Est, France). He is currently a Professor and the Head of the Laboratory “Ion-exchange membranes and processes” of the Membrane Institute of Kuban State University (Krasnodar, Russia); he was awarded Honoris Causa at Montpellier University (Montpellier, France); he was an Associate Professor at Laval University (Quebec, Canada); he received the order of Officier des Palmes académiques, France; and he was the Laureate of the National Prize of the Russian Professorial Assembly “PROFESSOR OF THE YEAR 2022” in the nomination category “Chemical Sciences”.

According to Scopus, Victor Nikonenko is the co-author of 242 articles that have been cited over 7000 times, including more than 20 articles that were published in *Membranes*. Victor Nikonenko has 240 co-authors from 17 countries including Canada, China, Germany, Israel, Japan, Portugal, the Russian Federation, Saudi Arabia, South Korea and Tunisia. However, the largest number of scientific papers (more than 100) were written in collaboration with French researchers from the Institut de Chimie et des Matériaux Paris-Est, Université Paris-Est Créteil (UMR 7182 CNRS-UPEC) and Institut Européen des Membranes, Université de Montpellier (UMR 5635 ENSCM-UM-CNRS). This collaboration was initiated in 1994 by the Membrane Club of France. It became even more fruitful after the French–Russian International Associated Laboratory “Ion-exchange Membranes and Related Processes” (Laboratoire International Associé Franco-Russe “Membranes échangeuses d’ions et procédés associés”) was created in 2011. The laboratory was organized within the framework of intergovernmental agreements between France and Russia. The co-directors of this laboratory were Prof. Victor Nikonenko, Prof. Gérald Pourcelly, Prof. Marc Cretin and Prof. Lasâad Dammak.

The international team has completed more than 20 scientific projects. The most significant projects were FP6 (European Commission, EC)—INTAS Kazakhstan (1997–2007), FP7 (EC) MemBridge (2009–2011), FP7 (EC, Marie Curie Actions) CoTraPhen 269135 (2011–2015), N° 38200SF Joint French–Russian PHC Kolmogorov Project (2017–2019), etc. These projects brought together scientific efforts and established good friendships between scientists and industrial partners from 20 countries. 

Professor Victor Nikonenko has become a mainstay of the scientific and international communities. His numerous collaborative scientific projects with European, American and Asian groups took various forms and were successful. Thanks to him, a large number of young people were trained. Many professional careers were shaped by his advice and unrivaled scientific generosity. Significant scientific achievements would not have been possible without his strong participation. Multilateral and transcontinental cooperation could not have started without his help and contribution.

To express our sincere gratitude and consideration to this scientific giant, this Special Issue is edited in honor of our friend, colleague, and master, Victor Nikonenko. We extend many thanks and a huge bravo for all that he has done for our community.

The scope of this Special Issue is as wide as the scientific horizons of Victor Nikonenko, including but not limited to:

- The mathematical modeling of the relationships between the structure–transport properties of membranes and of the transfer and selectivity phenomena in an electric field or in its absence;
- Commercial, experimental and modified ion exchange membranes (monopolar, bipolar, mosaic, composite, multilayer, organic, inorganic, homogeneous, heterogeneous, etc.);
- Commercial, experimental and modified ion exchange membranes’ transport characteristics and structure–property relationships;
- Concentration polarization and coupled phenomena (water splitting, electroconvection, gravitational convection, etc.) occurring when an electric field is applied;
- Ion exchange membranes’ behaviors in various modules and processes (dialysis, electrodialysis, electrolysis, capacitive deionization, fuel cells, microfluidic devices, bioreactors, potentiometric sensors, etc.);
- Ion exchange membranes’ fouling and scaling and ways to counter these phenomena;
- New methods of studying the properties of ion exchange membranes and membrane systems;
- Novel areas of ion exchange material use, their application in hybrid and combined membrane technologies of the circular economy, etc.

Georgy S. Ganchenko et al. [1] presents theoretical and experimental investigations of the behavior of an electrolyte solution with three types of ions near an ion-selective microparticle with electrokinetically driven and pressure-driven flows. They found that an enriched region with a high salt concentration appears at the anode side of the ion exchange bead when an electric field is turned on. This phenomenon is governed by nonequilibrium electroosmosis. The visualization experiments showed a good qualitative correlation with the numerical simulations. These results could be used in the future for developing microdevices for the detection and preconcentration of high-molecular organic substances and fragments of native structures before chemical and medical analyses.

Aminat Uzdenova [2] proposes a new approach to the theoretical description of the direct current mode in electromembrane systems, which contain ion exchange membranes as well as diluted and enriched diffusion layers. The new approach does not require boundary conditions on the derivative of the potential drop. The essence of the approach is to replace Poisson’s equation in the Nernst–Planck–Poisson system with the equation for the displacement current. The simulation results show that the use of the Nernst–Planck–Poisson system reduces the duration of the calculations. The application of the new approach increases their accuracy.

Jinfeng He et al. [3] compared the conversion ratio, current efficiency and energy consumption in the electrodialysis production of diprotic malic acid from sodium malate using different membrane stack configurations. They were BP-A, BP-A-C and BP-C (BP, bipolar membrane; A, anion exchange membrane; C, cation exchange membrane). The authors show that the conversion ratio follows BP-C > BP-A-C > BP-A, the current efficiency follows BP-A-C > BP-C > BP-A and the energy consumption follows BP-C < BP-A-C < BP-A. They demonstrate that electrodialysis with bipolar membranes is a clean, highly efficient, low-energy-consumption and environmentally friendly process to produce diprotic malic acid. 

Elodie Khetsomphou et al. [4] fabricated hierarchical cation exchange membranes and investigated the impacts of their properties and crosslinking density on whey demineralization performances via electrodialysis. These data were compared to the CMX cation exchange membrane, which performs well in whey demineralization. A 70% demineralization of 18% whey solution was reached for the first time for hierarchical cation exchange membranes without any fouling observed, and with comparable performances to the CMX benchmark. These promising results suggest the possible application of the new membrane in the food industry.

Tatiana Eliseeva and Anastasiia Kharina [5] studied current–voltage curves and the transport characteristics of heterogeneous highly basic and strongly acidic ion exchange membranes, during the electrodialysis of solutions containing a heterocyclic amino acid (proline or tryptophan) and a strong electrolyte (NaCl). The authors concluded that when cation and anion exchange membranes have prolonged contact with proline and tryptophan solutions, water splitting is enhanced due to the sorption of amino acids on their surfaces. The higher hydrophobicity of tryptophan causes more intensive electroconvection in the diluted solution compared to proline. The fouling of the ion exchange membranes by heterocyclic amino acids is reversible. A decrease in the fluxes of mineral salt ions with an increase in the mass transfer of the amino acid was observed in the intensive current mode.

Veronika Sarapulova et al. [6] focused on the shape of the current–voltage curve and water splitting in the case of the pristine and modified heterogeneous cation exchange membrane MK-40 in NaCl or CaCl_2_ solutions. The modification was carried out via the layer-by-layer method using the LF4-SK dispersion in isopropanol (analogous to Nafion), sodium polystyrene sulfonate (PSS), polyallylamine (PAN) or polyethylenimine (PEI). The film LF4-SK, which covers the modified membrane, homogenizes its surface. The authors of the article draw the readers’ attention to the different effects of PAN and PEI on the resistance of membrane systems in an applied electric field, as well as to the differences in the development of water splitting in the case of NaCl and CaCl_2_ solutions. These results are of interest to researchers who are trying to increase the selectivity of cation exchange membranes to monovalent cations.

Olga Kozaderova et al. [7] analyzed the patterns of the transport of ammonium and potassium counterions in the heterogeneous cation exchange membranes MK-40 and MK-41. To analyze the experimental concentration dependences of conductivity, the current–voltage curves of the membranes are used, the pH of their internal solution is determined and the electrodialysis treatments of the NH_4_NO_3_ and KNO_3_ solutions are carried out. In addition, a mathematical model that takes into account the cation exchange membrane and the diffusion layers adjacent to it is used to evaluate mass transfer coupled with proton transfer reactions between NH_4_^+^ and NH_3_·H_2_O species. Based on these data, the authors propose a specific mechanism for the transfer of NH_4_^+^ species to NH_3_·H_2_O species in a cation exchange membrane and explain the increase in the proton flux in ammonium-containing solutions, which is observed in the practice of electrodialysis.

Anna Parshina et al. [8] report on the development of a potentiometric multisensory system for the analysis of sulfamethoxazole and trimethoprim combination drugs. Perfluorosulfonic acid membranes containing functionalized CNTs were used as the sensor materials. The article considers the influence of the concentration of CNTs and their surface properties, as well as the influence of the preliminary ultrasonic treatment of the polymer and CNT solution before casting hybrid membranes upon their ion exchange capacity, water uptake and transport properties.

Polina Pushankina et al. [9] obtained thin Pd-40%Cu films using the classical melting and rolling method and magnetron sputtering. The films were modified via electrodeposition according to the classical method of obtaining palladium black and “Pd-Au nanoflowers” with spherical and pentagonal particles, respectively. The experiment results demonstrate that Pd-40%Cu films with a pentagonal structured coating have the highest catalytic activity, good resistance to CO fouling and long-term stability. The investigation of the developed membranes in the hydrogen transport processes in the temperature range of 25–300 °C also demonstrated a 1.5-time increase compared to membrane materials with classic niello. For all-metal modified membranes, the flux was increased by up to seven times compared to a smooth pure palladium membrane.

In their article, Denis Terin et al. [10] discuss the creation of reinforced ion exchange polymeric materials based on thermosetting matrices and high-temperature fibers with increased mechanical strength and chemical resistance. An original method for obtaining heterogeneous anion exchange composite membranes with the low-temperature ion plasma pretreatment of a polyester fibrous system is proposed. The influence of this pretreatment on the changes in the hydrophilicity, capillarity and structure of heterogeneous anion exchange composite Polykon A materials is studied.
